# Consumption of Different Egg-Based Diets Alters Clinical Metabolic and Hematological Parameters in Young, Healthy Men and Women

**DOI:** 10.3390/nu15173747

**Published:** 2023-08-27

**Authors:** Catherine J. Andersen, Lindsey Huang, Fangyi Zhai, Christa Palancia Esposito, Julia M. Greco, Ruijie Zhang, Rachael Woodruff, Allison Sloan, Aaron R. Van Dyke

**Affiliations:** 1 Department of Biology, Fairfield University, Fairfield, CT 06824, USA; julia.greco1@student.fairfield.edu (J.M.G.); allison.sloan@student.fairfield.edu (A.S.); 2 Department of Nutritional Sciences, University of Connecticut, Storrs, CT 06269, USA; lindsey.huang@uconn.edu (L.H.); fangyi.zhai@uconn.edu (F.Z.); ruijie.zhang@uconn.edu (R.Z.); rachael.woodruff@uconn.edu (R.W.); 3 Marion Peckham Egan School of Nursing and Health Studies, Fairfield University, Fairfield, CT 06824, USA; christa.esposito@fairfield.edu; 4 Department of Chemistry and Biochemistry, Fairfield University, Fairfield, CT 06824, USA; avandyke@fairfield.edu

**Keywords:** eggs, diet composition, body composition, metabolic panel, insulin sensitivity, serum lipids, lipoprotein profiles, clinical immune profiles, combined oral contraceptives

## Abstract

Eggs—particularly egg yolks—are a rich source of bioactive nutrients and dietary compounds that influence metabolic health, lipid metabolism, immune function, and hematopoiesis. We investigated the effects of consuming an egg-free diet, three egg whites per day, and three whole eggs per day for 4 weeks on comprehensive clinical metabolic, immune, and hematologic profiles in young, healthy adults (18–35 y, BMI < 30 kg/m^2^ or <30% body fat for men and <40% body fat for women, n = 26) in a 16-week randomized, crossover intervention trial. We observed that average daily macro- and micronutrient intake significantly differed across egg diet periods, including greater intake of choline during the whole egg diet period, which corresponded to increased serum choline and betaine without altering trimethylamine *N*-oxide. Egg white and whole egg intake increased serum isoleucine while whole egg intake reduced serum glycine—markers of increased and decreased risk of insulin resistance, respectively—without altering other markers of glucose sensitivity or inflammation. Whole egg intake increased a subset of large HDL particles (H6P, 10.8 nm) and decreased the total cholesterol:HDL-cholesterol ratio and % monocytes in female participants using combined oral contraceptive (COC) medication (n = 11) as compared to female non-users (n = 10). Whole egg intake further increased blood hematocrit whereas egg white and whole egg intake reduced blood platelet counts. Changes in clinical immune cell counts between egg white and whole egg diet periods were negatively correlated with several HDL parameters yet positively correlated with measures of triglyceride-rich lipoproteins and insulin sensitivity. Overall, the intake of whole eggs led to greater overall improvements in micronutrient diet quality, choline status, and HDL and hematologic profiles while minimally—yet potentially less adversely—affecting markers of insulin resistance as compared to egg whites.

## 1. Introduction

Eggs are a rich source of bioactive nutrients and dietary compounds with roles in the regulation of metabolic health, lipid metabolism, immune function, and hematopoiesis [1,2,3]. The composition of egg white and egg yolk fractions are distinct [4]. While high biological value protein and B vitamins are provided by both egg whites (10.9 g protein/100 g) and egg yolks (15.9 g protein/100 g), egg yolks additionally serve as a relatively rich source of choline-containing glycerophospho- and sphingolipids, fatty acids (34.7% saturated, 48.7% monounsaturated, and 16.7% polyunsaturated), water-soluble forms of choline, cholesterol, all vitamins except vitamin C—including the majority of B vitamins that are also present in lower amounts in egg whites. Eggs are additionally rich in minerals and essential trace elements, with egg yolks providing more grams of calcium, copper, iodine, iron, manganese, phosphorous, selenium, and zinc per 100 g, whereas egg whites are relatively richer in magnesium, potassium, and sodium [5]. Further, egg yolks contain antioxidant carotenoids, including lutein and zeaxanthin [6]. Accordingly, eggs are recognized as the most cost-effective animal source of protein and numerous micronutrients, which may reduce disease burden of at-risk and underserved populations. Egg consumption is additionally associated with greater overall nutrient density and quality of the diet [7,8,9].

Given the potential nutritional benefits and socioeconomic accessibility of egg intake, the effects of egg consumption on clinical measures of nutritional status, cardiometabolic risk factors, and hematologic (mainly erythrocyte) profiles have been evaluated in observational and intervention studies; however, the results are often inconsistent and controversial, which may in part be due to differences in study designs (number of eggs per day, duration of egg treatment), populations (healthy vs. metabolic dysfunction), background diets (weight stable vs. weight loss), and individual variability between subjects [10,11,12,13,14,15,16]. Further, studies often focus on a narrow range of biomarkers rather than providing a comprehensive assessment of standard clinical metrics, which is essential for determining the balance of potential beneficial vs. adverse responses to diet. As a result, studies have reported varied changing and null effects on serum lipids, lipoprotein profiles, and serum inflammatory markers [1,17] in addition to conflicting effects on serum trimethylamine *N*-oxide (TMAO) [18,19]—a pro-atherogenic choline-derived metabolite produced by gut bacteria [20]—despite recent epidemiolocal studies finding null or beneficial associations between egg intake and cardiovascular disease (CVD) outcomes [14,21]. Similarly, while epidemiological studies have reported conflicting associations between egg intake and type 2 diabetes mellitus (T2DM) risk [15,22], intervention studies often report a null or beneficial effect of egg intake on traditional markers of insulin resistance [10,19,23] and do not incorporate emerging biomarkers, such as plasma concentrations of branched-chain amino acids and glycine [24,25]. Further, studies report minimal to no effects of egg intake on improving anthropometric and erythrocyte profiles [16,26,27] and fail to incorporate an assessment of clinical immune profiles—despite the utility of white blood cell (WBC) and differential leukocyte counts in predicting chronic disease risk [28,29] and evidence from animal and human studies suggesting that egg intake alters immune cell gene expression, responsiveness to lipopolysaccharide, and pathogen defenses [30,31,32,33].

Therefore, we sought to evaluate the effects of consuming an egg-free, egg white, and whole egg diet on comprehensive clinical markers of diet quality, nutritional status, cardiometabolic risk, and hematologic profiles that are commonly assessed in healthcare settings and are controversial and/or lacking assessment in dietary intervention trials. We conducted this trial in young, healthy adults to evaluate diet effects without the complication of metabolic dysfunction or additional background diet confounders. In a subgroup analysis, we further evaluated whether the use of combined oral, hormone-based contraceptive (COC) medication in female subjects impacted the response to egg diets, given that COC use is prevalent in groups representative of our study population (young women aged 18–35 y), and the use of COC has been shown to impact cardiometabolic and immune measurements assessed in this study [34,35,36,37].

## 2. Materials and Methods

### 2.1. Study Participants

Twenty-eight women and men were recruited and enrolled to participate in a 16-week randomized cross-over dietary intervention trial. Participants were eligible for the study if they were age 18–35-years-old at the time of screening, had a BMI < 30 kg/m^2^ or <30% body fat for men and <40% body fat for women, and were willing to consume eggs and egg whites on a daily basis during study periods. Individuals were excluded from the study if they had a self-reported history of diabetes mellitus, coronary heart disease, stroke, renal problems, liver disease, cancer, autoimmunity, chronic infections, egg allergy, or current pregnancy or lactation. Additional exclusion criteria included taking lipid-lowering medications (e.g., statins, fibrates) and having a preexisting medical condition or implanted medical device that prevents participation in bioelectrical impedance measurements of body composition. Individuals were additionally excluded if they had fasting triglyceride levels higher than 500 mg/dL, fasting glucose higher than 126 mg/dL, and plasma total cholesterol greater than 240 mg/dL. Information on health history and use of medications and/or dietary supplements was collected via a medical questionnaire, whereas fasting blood lipid and glucose levels were measured in blood obtained via finger prick utilizing a Cholestech LDX analyzer and Lipid Profile + Glucose cassettes (Alere, Inc., Hayward, CA, USA) at screening. Body mass index (kg/m^2^) was calculated following measurement of body height and weight. Body height was determined utilizing a Tanita HR-200 wall-mounted height rod (Tanita Corporation of America, Arlington Heights, IL, USA), whereas body weight and body fat composition was measured utilizing a Tanita SC-240 digital scale with bioelectrical impedance function. This study was approved by the Fairfield University Institutional Review Board (protocol #0511), and all subjects provided written, informed consent prior to screening. This study was registered at ClinicalTrials.gov (NCT03577223). 

### 2.2. Study Design and Dietary Intervention

The 16-week dietary intervention utilized a randomized cross-over design. Following enrollment into the study, participants entered a 4-week egg-free diet run-in period, during which they were asked to refrain from consuming whole eggs, egg whites, or predominantly egg-based foods where egg fractions constituted more than a minor ingredient (egg-free diet). Participants were then randomly assigned to consume either 3 large shelled whole eggs/day (whole egg diet) or the equivalent of 3 large egg whites (0.5 cup liquid egg whites, Wholesome Farms, Sysco Corporation, Houston, TX, USA)/day (egg white diet) for 4 weeks. Participants then entered a 4-week egg-free washout period, followed by assignment to the alternative whole egg- or egg white-based diet treatment for the final 4 weeks of the intervention. All whole eggs and egg white food products were pasteurized and provided to the participants, who were asked to refrain from consuming any outside egg products. Whole eggs were provided as shelled eggs to provide participants with greater flexibility in preparation during the whole egg diet period, and 100% egg whites were provided in cartons during the egg white diet period. Egg white ingredients included egg whites, guar gum, and triethyl citrate as whipping aids. Participants were allowed to consume study eggs at any time of day using preferred cooking methods and instructed to fully cook eggs for food safety. Table 1 summarizes the difference in nutrition composition for each daily egg treatment (equivalent to 3 large whole eggs or 3 large egg whites per day).

Participants were asked to maintain their habitual dietary patterns, level of physical activity, and usage of supplements and medications throughout the duration of the study, all of which were monitored by surveys. Of note, approximately half of the female participants were taking combined oral contraceptive (COC) medication (n = 11, n = 10 non-users). Participants were weighed using a digital scale every two weeks to ensure weight maintenance throughout the intervention. 

**Figure 1:**
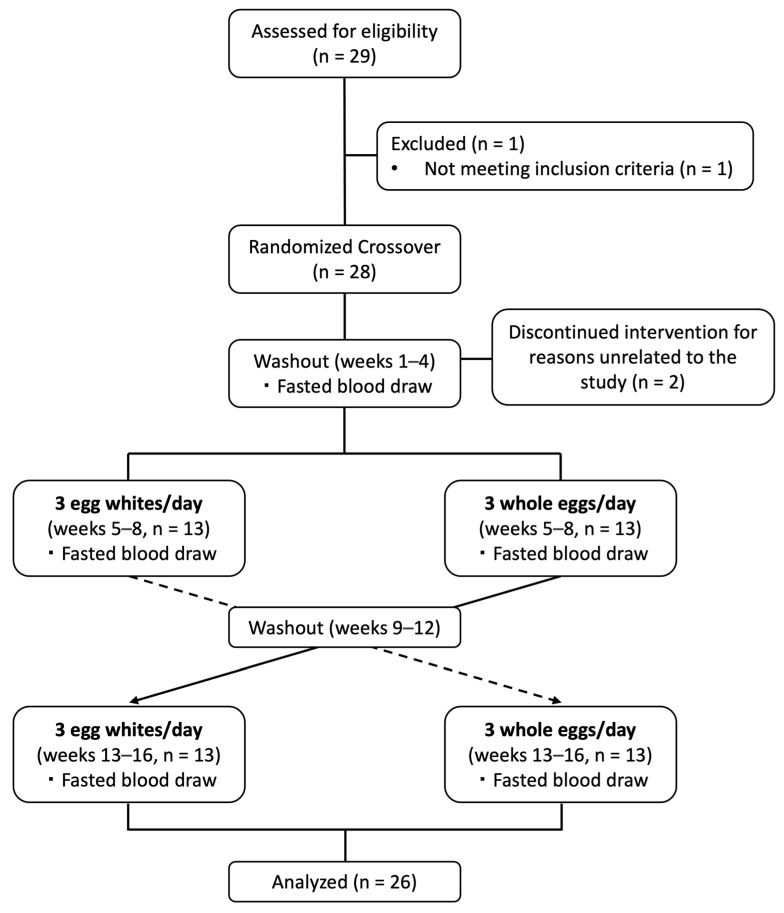
Diagram of study design and subject recruitment, enrollment, and completion.

A total of 29 individuals were screened for participation in the study. One individual did not meet the study criteria and was excluded from participating in the study. Twenty-eight participants (twenty-one female, seven male) met the study criteria and were enrolled into the study. Two participants (both male) withdrew for reasons unrelated to the study within the first few weeks of the egg-free diet period and were excluded from analyses. An overview of the study flowchart is presented in Figure 1.

### 2.3. Dietary Intake Analysis

To assess dietary nutrient and food group composition and compliance to egg treatments, participants completed 5-day food and beverage intake records at the end of the first egg-free diet period (week 4), and at the end of the egg white and whole egg diet periods (week 8 and week 16). The 5-day diet recording period consisted of 3 weekdays and 2 weekend days. Average daily nutrient composition and intake analysis was determined utilizing the Nutrition Data System for Research (NDSR, Nutrition Coordinating Center, University of Minnesota, Minneapolis, MN, USA). Compliance to the egg diet treatments was additionally monitored by completion of a daily egg intake questionnaire.

### 2.4. Body Composition Analysis

Body composition was determined at the end of the first egg-free diet period (week 4), and the end of the egg white and whole egg diet periods (week 8 and week 16). Body weight and composition (% body fat, fat mass, fat-free mass, muscle mass) were determined as described above utilizing a Tanita SC-240 digital scale with bioelectrical impedance function. BMI (kg/m^2^) was calculated following measurement of body height and weight, with body height determined as described above utilizing a Tanita HR-200 wall-mounted height rod (Tanita Corporation of America, Arlington Heights, IL, USA).

### 2.5. Blood Collection

Fasted blood samples were collected after a 12-h overnight fast via venipuncture into EDTA and SST tubes at the end of the first egg-free diet period and the end of the whole egg and egg white diet periods. Serum was isolated by centrifugation at 1200× *g* for 10 min at 20 °C in an Allegra X-14R swing-bucket centrifuge (Beckman Coulter, Inc., Brea, CA, USA) and aliquoted under sterile conditions for analyses described below. EDTA whole blood was aliquoted for analysis of complete blood cell counts.

### 2.6. Clinical Metabolic Parameters and Measures of Choline Status

Clinical metabolic profiles were determined in fasting serum. Fasting serum glucose and lipids, including total cholesterol, HDL-cholesterol (HDL-C), and triglycerides, were measured by spectrophotometry by Quest Diagnostics (Seacaucus, NJ, USA). LDL-cholesterol (LDL-C) was determined using the Martin–Hopkins calculation. Serum electrolytes (sodium, potassium, chloride, calcium), carbon dioxide, measures of protein status, liver, and kidney function (blood urea nitrogen, serum total protein, albumin, globulin, albumin:globulin ratio, bilirubin, alkaline phosphatase), and the acute-phase liver enzymes alanine aminotransferase (ALT) and aspartate aminotransferase (AST) were additionally measured by Quest Diagnostics. High-sensitivity C-reactive protein (hsCRP) was measured enzymatically utilizing a Cobas c-111 clinical analyzer (Roche Diagnostics, Florham Park, NJ, USA). Serum concentrations of ketones (total ketone bodies, beta-hydroxybutyrate, acetoacetate, and acetone), choline, betaine, and TMAO were measured by LabCorp (Morrisville, NC, USA).

### 2.7. Lipoprotein Size Profiles

Triglyceride-rich lipoprotein (TRLP), LDL, and HDL particle profiles were determined by nuclear magnetic resonance (NMR) by LabCorp (Morrisville, NC, USA) in serum samples, as previously described [10]. Measures included the concentration of total TRLP, LDL, and HDL particles, in addition average TRLP, LDL, and HDL particle diameters (nm). Serum concentrations particle subclasses were additionally reported, including concentrations of very large TRLP (90–240 nm), large TRLP (50–89 nm), medium TRLP (37–49 nm), small TRLP (30–36 nm), and very small TRLP (24–29 nm). For LDL, serum concentrations of large LDL (21.5–23 nm), medium LDL (20.5–21.4 nm), and small LDL (19–20.4 nm) were reported. HDL subclasses are reported as the concentrations of total small (7.4–8.0 nm), medium (8.1–9.5 nm), and large (9.6–13 nm) HDL in addition to concentrations of HDL particles of defined sizes: H1P (7.4 nm), H2P (7.8 nm), H3P (8.7 nm), H4P (9.5 nm), H5P (10.3 nm), H6P (10.8 nm), H7P (12.0 nm). Data for TRLP and LDL are reported as nmol/L, whereas data for HDL are reported as μmol/L. Serum concentrations of apolipoprotein B (apoB) and apolipoprotein A-1 (apoA-1) were similarly measured by NMR and are reported as mg/dL.

### 2.8. Serum Amino Acid and Insulin Resistance Measures

Serum concentrations of amino acids (total branched-chain amino acids, valine, leucine, isoleucine, alanine, and glycine) were measured by LabCorp using liquid chromatography/tandem mass spectrometry (LC-MS/MS) analysis. Serum glycA—a measure of protein levels and glycosylation states of several of the most abundant acute-phase proteins in serum [39]—and citrate were determined by NMR. Data generated from lipoprotein profiles and amino acids were additionally used to calculate (1) a lipoprotein–insulin resistance index (LP–IR), and (2) diabetes risk index (DRI), as reported by LabCorp. LP–IR is calculated from a combination of six lipoprotein parameters, including large very low-density lipoprotein (VLDL) particle number, VLDL size, small low-density lipoprotein (LDL) particle number, LDL size, large high-density lipoprotein (HDL) particle number, and HDL size via LabCorp’s proprietary Vantera^®^ platform (Coral Gables, FL, USA). LP–IR values range from 0 (most insulin sensitive) to 100 (most insulin resistant). DRI is calculated from LP–IR plus the concentrations of valine and leucine. DRI values range from 0 (most insulin sensitive) to 100 (most insulin resistant).

### 2.9. Complete Blood Cell Counts

Complete blood cell counts were measured in freshly collected EDTA whole blood by Quest Diagnostics by electronic cell sizing/counting/cytometry/microscopy. Data reported include total white blood cell counts and differential absolute and % neutrophils, lymphocytes, monocytes, eosinophils, and basophils. Additional hematologic parameters reported include total red blood cells, total hemoglobin and hematocrit, mean corpuscular volume and mean corpuscular hemoglobin concentration, and platelet concentrations. Clinically-relevant immune ratios, including neutrophil:lymphocyte and lymphocyte:monocyte ratios were calculated as follows: neutrophil:lymphocyte ratio = absolute neutrophils ÷ absolute lymphocytes; lymphocyte:monocyte ratio = absolute lymphocytes ÷ absolute monocytes [40,41].

### 2.10. Statistical Analysis

Analysis was performed on data derived from the 26 participants who completed the study unless otherwise noted due to limited sample availability to complete all measures for each participant. Baseline characteristics of participants included in final analyses are included in Supplementary Table S1. Samples from participants at each time point were analyzed in the same batches in order to minimize variability across assay runs. Repeated measures ANOVA with pairwise comparisons was performed using SPSS Version 28 to compare the effects of different egg diets on clinical outcome measures. Independent *t*-tests were used to compare changes in outcome measures following the egg white vs. whole egg period between COC users (n = 11) vs. non-users (n = 10) in female participants. Bivariate Pearson correlations were performed to evaluate associations between changes in metabolic and hematologic parameters across diet periods. All data are presented as mean ± standard deviation unless otherwise noted. Values with different letters (a, b, c) are significantly different, as are comparisons denoted with an asterisk (* *p* < 0.05).

## 3. Results

### 3.1. Average Daily Nutrient Intake Differed across Egg Diet Periods

Given the differences in the nutrient composition of egg whites and egg yolks, we first evaluated whether adherence to the different egg diets impacted average daily nutrient intake of the diet as a whole. An analysis of five-day dietary records indicated that an average daily intake of nutrients with known anti-inflammatory, antioxidant, metabolic, immunomodulatory, and hematopoietic properties differed across egg diet periods (Table 2). While energy intake remained consistent across diet periods, % macronutrient intake differed, with % carbohydrate and % fat intake decreasing and increasing, respectively, from the egg-free diet to the egg white and whole egg diets. Compared to the egg-free diet, intake of animal protein, polyunsaturated fat, and sodium was increased during the whole egg diet, whereas intake of total fat, monounsaturated fat, arachidonic acid, and cholesterol was increased during the whole egg diet period compared to both the egg-free and egg white diet periods. Consumption of total protein foods and oils on average were increased during the egg white and whole egg diet periods as compared to the whole egg diet period (Table S2). We additionally assessed the intake of amino acids that serve as serum indicators of insulin resistance, which were not changed over the course of the intervention. In line with egg yolks serving as a rich, bioavailable source of the antioxidant carotenoids lutein and zeaxanthin [4,6], we observed that, compared to the egg-free diet, the average daily intake of lutein and zeaxanthin was increased during the whole egg diet period. Notably, intake of various micronutrients was also increased in the whole egg diet periods relative to the egg white diet period, including pantothenic acid, vitamin B12, vitamin D, and phosphorus, whereas selenium intake was greater during the whole egg diet period compared to both the egg-free and egg white diet periods. Finally, given that egg yolks serve as a rich source of choline (provided by glycerophospho- and sphingolipids) [11], average dietary intake of choline was increased during the whole egg diet period compared to both the egg-free and egg white diet periods.

### 3.2. Effect of Egg Intake on Body Composition and Clinical Metabolic Profiles

We next evaluated the effects of egg intake on body composition, measures of protein and choline status, kidney and liver function, and inflammation—including the acute-phase liver enzymes ALT, AST, and hsCRP (Table 3). Despite monitoring and dietary guidance to ensure weight maintenance throughout the diet periods, body weight increased by 0.9% on average during the egg white period as compared to the egg-free diet period, although this did not equate to significant changes in fat, fat-free, or muscle mass. In contrast, while body weight did not significantly differ between the egg-free and whole egg diet period, fat mass was increased by 3.5% on average following the whole egg diet. In assessing metabolic panels, we did not observe differences in markers of protein status, kidney and liver function, inflammation, or ketone concentrations across diet periods. However, we observed significantly higher concentrations of serum choline and the choline metabolite betaine following the whole egg period, in line with increased dietary choline intake during this intervention phase. Notably, serum concentrations of TMAO were not altered between egg diet periods.

### 3.3. Effects of Egg Intake on Serum Lipid Profiles

**Figure 2:**
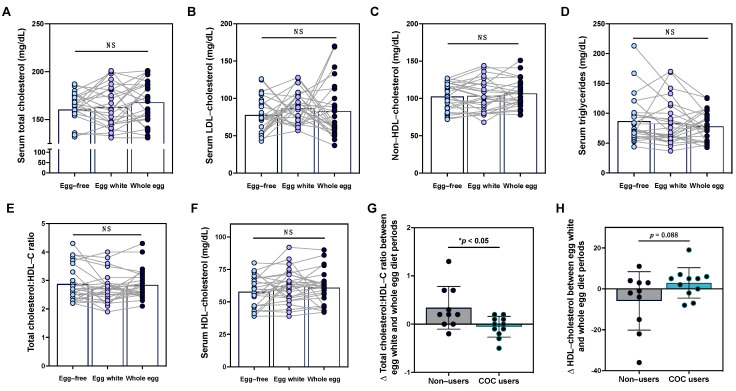
Serum lipid responses to egg intake are altered in females based on used of combined oral contraceptives. Intake of an egg-free diet (light blue), egg whites (purple), or whole eggs (dark blue) did not alter serum (**A**) total cholesterol, (**B**) LDL-C, (**C**) non-HDL-C, (**D**) triglycerides, (**E**) total cholesterol:HDL-C ratio, or (**F**) HDL-C in young healthy men and women (n = 26). In female participants, changes in the total cholesterol:HDL-C ratio (**G**) and (**H**) HDL-C between the egg white and whole diet periods were evaluated in COC users (teal bar, n = 11) as compared to non-users (gray bar, n = 10). * *p* < 0.05, NS: non-significant.

We next evaluated the effects of egg intake on serum lipid profiles. While there was notable variability across individuals, changes in fasting total cholesterol, LDL-C, HDL-C, non-HDL-C, triglycerides, or the total cholesterol:HDL-C ratio did not reach statistical significance (Figure 2A–F), with no differences observed between male and female participants. Interestingly, in conducting a subgroup analysis of female participants, we observed that variability in serum lipid responses to egg diets may in part be attributable to the use of combined oral contraceptives (COCs). Notably, changes in total cholesterol:HDL-C ratio between the egg white and whole diet periods were significantly decreased in COC users as compared to non-users (Figure 2G). This effect appeared to be primarily driven by changes in HDL-C between COC users vs. non-users, although differences did not reach statistical significance (Figure 2H).

### 3.4. Effects of Egg Intake on Lipoprotein Particle Profiles

**Figure 3:**
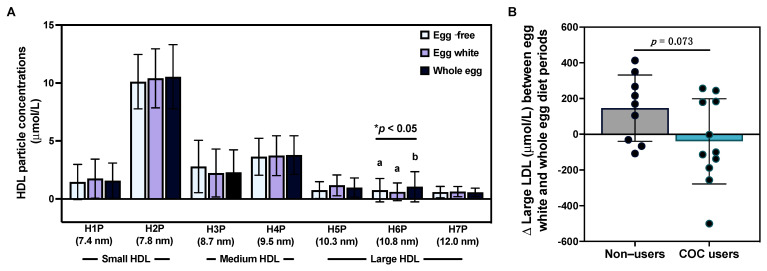
Effects of egg intake on serum lipoprotein profiles. Intake of an egg-free diet (light blue), egg whites (purple), or whole eggs (dark blue) on (**A**) HDL particle subclasses (n = 25); (**B**) changes in large LDL concentrations between the egg white and whole diet periods in female COC users (teal bar, n = 11) as compared to non-users (gray bar, n = 9). * *p* < 0.05. Different letters (a, b) denote statistically significant comparisons.

Despite observing only minimal effects of egg intake on serum lipids—and only in female participants based on COC use—we further evaluated whether the intake of different egg diets impacted lipoprotein particle profiles. Overall, we did not observe changes in average TRLP, LDL, or HDL particle size; serum concentrations of total TRLP, LDL, or HDL particles; apoB or HDL-associated apoA-1; and size subclasses of TRLP, LDL, and total large, medium, and small HDL (Table S3). However, we observed that HDL H6P (10.8 nm)—a subclass of large HDL—was increased following the whole egg diet as compared to the egg-free and egg white diet periods (*p* < 0.05; Figure 3A). Similar to differences in total cholesterol:HDL-C in response to egg intake observed between female COC users vs. non-users, we observed a trend toward greater increases in large LDL concentrations following the whole egg diet period in non-users (Figure 3B).

### 3.5. Effects of Egg Intake on Markers of Insulin Sensitivity

We next evaluated the effects of egg intake on fasting glucose and markers of insulin resistance, given that some epidemiological studies have reported associations between egg intake and type 2 diabetes mellitus risk, although these findings are not consistent across studies [15,42,43,44,45]. In line with previous intervention studies [10,23,46], we did not observe changes in fasting glucose across diet periods (Table 4. Further, we did not observe changes in glycA—a measure of protein levels and glycosylation states of several of the most abundant acute-phase proteins in serum [39]. Given recent reports that serum amino acid levels have predictive potential in evaluating T2DM risk [24,25], we next evaluated whether intake of different egg-based diets altered serum amino acid profiles. Interestingly, we observed that serum isoleucine was increased in the egg white and whole egg diet periods as compared to the egg-free diet period, whereas serum glycine was increased following the whole egg diet only. While serum concentrations of branched-chain aminos are positively correlated with insulin resistance [24], glycine, a conditionally essential amino acid, circulates at a lower level in T2DM patients as compared to healthy controls [25], suggesting a greater potential protective effect of whole eggs against T2DM as compared to egg whites. Despite these differences, no changes in lipoprotein insulin resistance index (LP–IR) or diabetes risk index (DRI)—which take into account lipoprotein profiles and serum amino acid concentrations to characterize insulin resistance and T2DM risk [47,48]—were observed across diet periods.

### 3.6. Effects of Egg Intake on Clinical Immune Profiles

**Figure 4:**
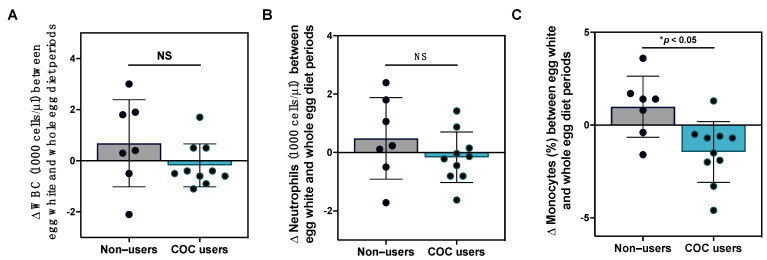
Effects of egg intake on clinical immune profiles with COC use. Changes (**A**) total WBC; (**B**) absolute neutrophils; and (**C**) % monocytes between the egg white and whole diet periods in female COC users (teal bar, n = 10) as compared to non-users (gray bar, n = 7). * *p* < 0.05, NS: non-significant.

Previous studies have reported changes in systemic inflammatory markers and peripheral blood mononuclear cell inflammatory responsiveness following egg intake [11,30,49]; however, the effects of egg intake on clinical immune profiles are less well-characterized. In this current study, we did not observe statistically significant changes in total white blood cell or differential leukocyte counts between egg diet periods, nor did we observe changes in the percent distribution of leukocyte subsets, although notable variability in responses was observed (Table S4). We further evaluated the effects of egg intake on clinical leukocyte ratios that have prognostic potential in chronic inflammatory conditions, including neutrophil:lymphocyte and lymphocyte:monocyte ratios [40,50,51]; however, these metrics did not change over the course of the intervention. Given that serum lipid responses to diet differed in female participants with COC use, we further evaluated whether COC use dictated the effects of egg intake on clinical immune markers. While changes in total WBC and neutrophil counts between the egg white and whole egg diet periods with COC use did not reach statistical significance, we observed that changes in % monocytes between egg white and whole egg periods were significantly reduced in COC users as compared to non-users (Figure 4).

### 3.7. Effects of Egg Intake on Clinical Erythrocyte and Platelet Profiles

**Figure 5:**
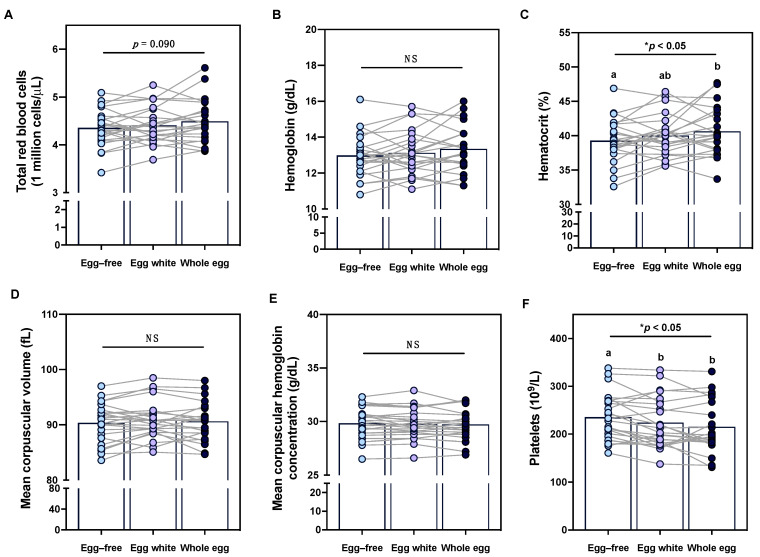
Effects of egg intake on clinical erythrocyte and platelet profiles. Intake of an egg-free diet (light blue), egg whites (purple), or whole eggs (dark blue) on (**A**) total red blood cells; (**B**) hemoglobin; (**C**) hematocrit; (**D**) mean corpuscular volume; (**E**) mean corpuscular hemoglobin concentrations; and (**F**) total platelets (n = 23). Different letters (a, b) denote statistically significant comparisons. * *p* < 0.05, NS: non-significant.

In addition to evaluating the effects of egg intake on immune profiles, we further assessed whether intake of different egg-based diets altered erythrocyte markers—particularly given the changes in reported dietary intake of hematopoietic nutrients throughout the intervention. While changes in total red blood cell counts did not reach statistical significance, hematocrit increased following the whole egg diet as compared to the egg-free diet (Figure 5A,C). Conversely, no changes in hemoglobin, mean corpuscular volume, or mean corpuscular hemoglobin concentrations were observed between diet periods (Figure 5B,D,E). Interestingly, platelets decreased following both the egg white and whole egg diet period as compared to the egg-free diet period (Figure 5F). The effects of egg intake on erythrocyte and platelet measures did not differ in female participants with COC use—despite previous reports that COC use has been associated with higher hemoglobin and red blood cell and platelet counts [37].

### 3.8. Egg-Induced Changes in Lipoprotein and Glucose Sensitivity Measures Differentially Correlate with Changes in Immune Cell Subset Counts

**Figure 6:**
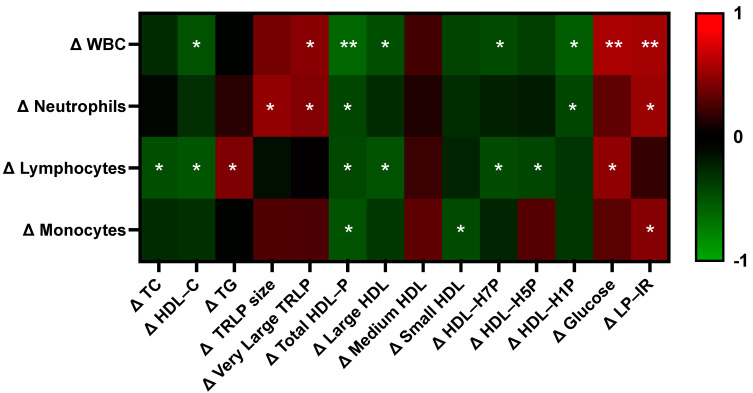
Correlations between changes in metabolic vs. immune parameters. Heatmap values are Pearson correlation coefficients, where red indicates a positive correlation and green indicates a negative correlation. Asterisks denotes statistically significant correlations (* *p* < 0.05, ** *p* < 0.01). HDL-P: HDL-particle, LP–IR: lipoprotein–insulin resistance index, TC: total cholesterol, TG: triglycerides, TRLP: triglyceride-rich lipoproteins, WBC: white blood cells.

Beyond the potential direct role of egg-derived nutrients on metabolic and hematologic outcome measures, we hypothesized that variable changes in immune and erythrocyte parameters across participants may additionally be a result of improved metabolic profiles—specifically the changes in the concentration of large HDL particles, as HDL has been shown to influence immune cell activation, differentiation, and erythrocyte lifespan [52,53,54,55]. In conducting statistical correlation analysis, we found that changes in specific serum lipid and lipoprotein subclasses differentially correlated with changes in total white blood cell, neutrophil, lymphocyte, and monocyte counts between egg white and whole egg diet periods (Figure 6). Notably, the greatest number of correlations were observed between HDL and immune measures, as compared to other serum lipid and lipoprotein fractions. Overall, changes in HDL measures negatively correlated with immune cell counts with the exception of medium HDL and with changes in the concentration of total HDL particles having the strongest overall relationship across immune cell types. Further, changes in fasting serum triglycerides, very large TRLP, glucose, and LP–IR were positively correlated with changes in different immune cell counts.

## 4. Discussion

Eggs are a rich source of bioactive nutrients and dietary compounds that possess properties that may contribute to the regulation of metabolic health, lipid metabolism, immune function, and hematopoiesis [1,2,3]. However, the effects of egg intake on biomarkers related to these health parameters are often inconsistent and evaluated in isolation, making it difficult to evaluate the comprehensive effects of egg intake on standard, routine clinical parameters that are more likely to influence a healthcare provider’s dietary recommendations [1,45]. In this study, we observed that intake of an egg-free diet, 3 egg whites per day, and 3 whole eggs per day altered the global dietary nutrient composition, with whole eggs leading to greater overall improvements in micronutrient diet quality. Further, whole egg consumption improved choline status and HDL, immune, and hematologic profiles while minimally—yet potentially less adversely—affecting markers of insulin resistance as compared to egg whites. Importantly, the use of COC by female participants altered the serum lipid and immune responses to egg diets, and correlation analysis suggests that egg-induced changes in HDL and insulin resistance markers correlated with changes in immune parameters while correlations between other metabolic and hematologic markers were not observed. Together, these findings suggest that, despite increases in body fat mass and certain nutrients associated with chronic disease pathophysiology [56,57], whole egg intake may confer greater global metabolic and hematologic benefits compared to egg-free and egg white diets, although effects may differ based on the use of hormone-based medication.

Despite being a relatively rich dietary source of cholesterol, whole egg consumption has been positively associated with greater overall diet quality, including greater daily intake of high-quality protein, polyunsaturated and monosaturated fat, α-linolenic acid, docosahexaenoic acid (DHA), vitamin D, potassium, phosphorus, selenium, choline, and the carotenoids lutein and zeaxanthin—the majority of which are provided by the egg yolk [2,4,8,58]. Similar dietary patterns were observed in our study, with greater enrichment of many of these nutrients during the whole egg period. Beyond the intake of egg yolk nutrients, egg consumers tend to incorporate more total protein foods, seafoods, total vegetables, whole fruits, whole grains, and dairy foods, leading to healthier whole food patterns [8,58]. Besides the benefit of greater diet quality, egg consumers reported higher postprandial satiety and suppressed ghrelin responses [23,59,60] as well as decreased intake of total and added sugar compared to non-egg consumers [58,61]. These findings are in line with the results from our study, in which the percent of kilocalories coming from carbohydrates was reduced in the whole egg diet period as compared to the egg-free and egg white diet periods while total energy intake across diet periods did not differ. It is important to note that the intake of various nutrients associated with cardiometabolic disease were increased by whole egg intake, including total fat, arachidonic acid, and sodium [56,57,62]. However, a greater intake of these nutrients did not correspond to adverse changes in cardiometabolic or hematologic profiles—despite minor changes in body composition.

Reports on the effects of egg intake on body weight and composition yield conflicting results from epidemiological and intervention studies. In a cross-sectional study by Garrido-Miguel et al. [26], consumption of ≥5 eggs/week was associated with lower BMI and body fat percentage compared to participants consuming <1 egg/week in a young adult population (age 18–30 years old), suggesting that greater egg consumption may promote a healthier body composition, which is in line with studies finding that eggs increase satiety and improve diet quality [8,23,58,59,60]. Interestingly, reductions of body weight following dried whole egg intake has also been observed in diabetic and diet-induced obesity rat models [63,64]. Population-based and observational studies have also reported conflicting sex-specific differences in the effects of egg intake on body composition, with consumption of >50 g egg/day being associated with reduced risk of central obesity and body fat in females only in a study of 2241 Chinese adults (age 18–80 y) [65], whereas stronger protective associations between egg intake and reduced risk of being classified as metabolically unhealthily obese were observed in males [66]. In contrast, Shim and Seo [27] observed that an intake of 2–3 eggs/week and 4–6 eggs/week based on food frequency questionnaire responses were associated with higher body fat mass in males and females, respectively, in 13,366 adults from the Korea National Health and Nutrition Examination Surveys 2008–2011. Variability in results across studies may be attributable to differences in study population characteristics (including age, sex, and health status), background dietary patterns, methods for assessing diet and body composition, and criteria for classifying high vs. low egg intake. Further, these results often do not translate to those observed in randomized, controlled intervention studies, in which an intake of 1–3 eggs/day has not been associated with changes in body weight or composition in healthy adults [19]. In our study, we observed a 0.9% increase in body weight and a 3.5% increase in fat mass in the egg white and whole egg diet period, respectively, as compared to the egg-free diet period, with no differences between males and females. However, it is unclear if these results are physiologically significant given a lack and/or improvement in metabolic profiles from whole egg intake, or whether these changes are artifacts or coincidental natural fluctuations in weight that would persist long-term. Additional intervention studies are warranted to elucidate whether certain dietary patterns or metabolic profiles are determinants of body composition change from egg intake.

In line with increased dietary choline intake observed during the whole egg period, serum concentrations of choline and the choline metabolite betaine were higher following the whole egg diet period, which is consistent with previous egg intervention studies [49,67]. Egg yolks serve as a rich source of choline (~125 mg per large egg), which is an essential component of cell membrane phospholipids, a precursor to the neurotransmitter acetylcholine; regulates one-carbon metabolism and homocysteine levels through the formation of betaine; and supports neural tube and brain development and cognitive function in early life [2,4,68]. Betaine has also been shown to possess anti-inflammatory activities through inhibition of nuclear factor-κB activity, NLRP3 inflammasome activation, and mitigation of endoplasmic reticulum stress [69]. Further, choline provided by egg yolk has been shown to be more bioavailable and efficiently absorbed compared to a choline bitartrate supplemented beverage [68]. Incorporating bioavailable sources of choline into the diet is essential, as choline intake by pregnant women, children, and adult men and women often fall far below Adequate Intake (AI) levels [70]. Through the analysis of >25,000 participants in the National Health and Nutrition Examination Survey 2005–2014 datasets, Wallace and Fulgoni [71] reported that egg consumers were found to have a higher intake of choline compared to non-egg consumers and concluded that the ability of individuals to meet the AI for choline is extremely difficult unless they consume eggs or a dietary supplement that provides choline. With the AI for men and women age 19+ being 550 mg and 425 mg/day, respectively [72], our study participants only met the daily AI for choline during the whole egg diet period on average (614.1 mg/day), whereas the average daily intake of choline during the egg-free (289.5 mg/day) and egg white (221.2 mg/day) diet periods fell below the AI. Importantly, we did not observe changes in serum TMAO—a proatherogenic choline metabolite produced by intestinal microbiota, which early studies linked to be increased by egg intake [18,73]. However, numerous intervention studies have since demonstrated that the intake of up to three whole eggs/day does not increase plasma TMAO in young healthy subjects [19,74,75], overweight postmenopausal women [67], or subjects with metabolic syndrome [76], which is consistent with our observations. Together, these findings suggest that whole intake supports improvements in choline status without adversely impacting choline-derived CVD risk factors.

The lack of effect of our study intervention on serum lipids and apoB-containing lipoprotein size profiles similarly suggests that cardiovascular disease biomarkers and risk are not adversely impacted by whole egg intake [77,78]. In contrast, the increase in HDL H6P—a subclass of large HDL—following the whole egg period is indicative of reduced CVD risk in population-based studies [3,79,80]. These findings are consistent with egg intervention studies conducted in young and older healthy adults as well as adults classified as overweight, with metabolic syndrome, and type 2 diabetes, with many studies additionally reporting increases in HDL-C [10,19,46,81,82,83]. The effects of whole egg intake on HDL parameters may in part be attributable to yolk-derived phospholipids, given that dietary phospholipid feeding has been shown to increase HDL-C in human and animal studies and that dietary phosphatidylcholine—the predominant phospholipid egg yolk—is preferentially incorporated into HDL particles following ingestion [84]. Egg intake has additionally been shown to increase lecithin cholesterol acyltransferase (LCAT) activity, which facilitates the esterification of free cholesterol to cholesteryl esters and promotes HDL maturation, stabilization, and increased particle size [10,19]. More recent studies have indicated diverse functions depending on HDL particle size; for instance, large particles have shown stronger antioxidant function [85,86] and were positively associated with HDL efflux capacity in 402 participants from the Chicago Healthy Aging Study [87]. Further research is warranted to evaluate the mechanisms by which egg intake modulates serum lipid and HDL profiles across individuals with varied and seemingly similar health phenotypes.

To explore potential factors that could influence the serum lipoprotein response to egg intake, we evaluated differences in serum lipid and lipoprotein profile changes in female subjects who were taking COCs vs. those who were not. Previous studies have shown mostly negative associations between COC use and serum lipid profiles; more specifically, various studies have shown that COC use is associated with significant increases in TC, LDL-C, and a reduction in HDL-C and the HDL-C/LDL-C ratio [34,35,36]. While COC use alters blood lipids, Momeni et al. [88] reported neutral effects on plasma levels of homocysteine and nitric oxide plasma levels in healthy young women. In our study, we observed greater increases in total cholesterol:HDL-C ratios in female subjects not using COCs as compared to COC users as well as trends toward decreases in HDL-C and increases in large LDL concentrations. In epidemiological studies, a shift from large LDL to greater concentrations of medium and small LDL particles has been associated with increased risk of CVD and coronary heart disease [89]. While these changes observed in our study did not reach statistical significance, it highlights an important consideration for conducting dietary intervention studies, particularly given that 19.5% of women age 15–19, 21.6% of women age 20–29, and 10.9% of women age 30–39 use COC [90]. Further research is warranted to determine whether COC and other hormone-based therapies influence physiological response to diet in intervention settings.

In addition to evaluating traditional cardiovascular disease risk factors, we evaluated the effects of egg intake on standard and emerging clinical biomarkers of insulin resistance. Epidemiological studies have reported conflicting results on the association between egg intake, markers of glucose control, and type 2 diabetes risk, with some studies reporting that higher daily intake of eggs is positively associated with higher blood glucose and greater risk of type 2 diabetes [15,91], whereas others have found that egg consumption is associated with lower risk of type 2 diabetes in middle-aged and old men in Korea [22]. In intervention trials, whole egg intake has not been shown to have neutral or beneficial effects on fasting glucose levels or insulin resistance markers (e.g., plasma insulin, HOMA-IR) in young healthy adults, subjects with metabolic syndrome, or type 2 diabetes [10,19,23,46]. Similarly, we did not observe changes in fasting glucose across egg diets in our study, nor did we observe changes in glycA—a measure of protein levels and glycosylation states of several of the most abundant acute-phase proteins in serum [39]. Interestingly, however, we observed that serum isoleucine was elevated in the egg white and whole egg diet periods as compared to the egg-free diet period, whereas serum glycine was elevated only following the whole egg diet. Reduced gene expression involved in branched-chain amino acid metabolism is one of the characteristics of type 2 diabetes [92]; thus, serum concentrations of branched-chain aminos are positively correlated with insulin resistance [24,25]. In contrast, serum glycine was found to be low in patients with obesity and diabetes [93,94], and an improvement of diabetes is positively associated with higher serum glycine level [95]. Our findings suggest that, perhaps, components of egg whites may be contributing to the positive associations between egg intake and insulin resistance, whereas components of egg yolk may counteract or balance these effects. A potential mechanism by which egg yolks can increase glycine concentrations is through the provision of choline, which is metabolized to betaine (or trimethylglycine) and ultimately glycine [25], which would correspond to our observations that serum choline and betaine were increased by whole egg intake.

We further evaluated the effects of egg intake on clinical immune profiles, for which there are limited studies, and serum inflammatory markers, for which studies have reported conflicting results [1,96]. In healthy individuals, whole egg intake has variable effects in serum inflammatory markers, including serum amyloid A and hsCRP, with studies reporting both pro-inflammatory and anti-inflammatory effects of egg intake [11,97,98,99]. However, reduction in plasma inflammatory markers from whole egg intake is more commonly observed in populations characterized by metabolic dysfunction and chronic low-grade inflammation (e.g., metabolic syndrome, overweight) [11,49,98]. While yolks provide a variety of nutrients with anti-inflammatory properties, a study by Dibella et al. [49] demonstrated that whole egg intake and choline bitartrate supplementation both decreased IL-6, whereas only whole eggs reduced hsCRP, suggesting that the choline content of whole eggs is in part attributable to their anti-inflammatory effects. Despite increasing serum concentrations of choline, whole egg intake did not significantly alter hsCRP, ALT, or AST in our study population, suggesting that further research is needed to identify participant characteristics or other determinants of the variable inflammatory responses to egg intake.

In addition to serum inflammatory markers, total white blood cell counts and differential immune subset counts can serve as important indicators of immune activation, function, and competence within the context of pathogen defense, infection, cancer, and autoimmunity [100,101,102]. Studies have shown that various clinical leukocyte parameters, including total white blood cells counts, absolute neutrophils and monocytes, and the ratio of neutrophils:lymphocytes and lymphocytes:monocytes are indicators of systemic inflammation and are positively associated with chronic metabolic diseases, such as metabolic syndrome and CVD [102,103,104]. In preclinical studies, whole egg and its lipid constituents have been shown to improve humoral immune status and restore T cell responsiveness, highlighting a potential modulatory role of egg intake in pathogen defense and clearance [32,33]. In clinical trials, egg intake has additionally been shown to reduce anti-inflammatory T regulatory and pro-inflammatory Th17 T cell counts, alter cytokine secretion from LPS-stimulated peripheral blood mononuclear cells, and improve clearance of active pulmonary tuberculosis from sputum cultures as part of a cholesterol-rich diet [30,31,99]. Consistent with the lack of effect on serum inflammatory markers, we did not observe changes in clinical immune parameters across diet periods in the study population as a whole, although whether our intervention induced changes in specific leukocyte subsets (e.g., Treg vs. Th17) and inflammatory responsiveness to stimuli that cannot be detected by measurement of traditional, clinical immune profiling warrants further study. Notably, in categorizing female subjects by COC use, we observed greater increases in % monocytes from the egg-free to egg white diet periods as compared to changes in % monocytes from the egg-free to whole egg diet periods, whereas changes in total WBC, absolute neutrophils, or other inflammatory markers did not reach statistical significance. Previous studies have shown that COC use is associated with an increase in inflammatory blood biomarkers including CRP, MCP-1, and pro-inflammatory cytokines, such as TNF and IL-6 [105,106]. More recently, a 2022 review by Tekle et al. [37] highlighted that, compared to non-users, COC users did not have significantly altered leukocyte counts across multiple studies. However, this review was a narrative review and, therefore, did not show the pooled effect of oral contraceptives on these parameters. While higher absolute monocyte counts have been associated with increased CVD risk [107], the clinical significance of changes in % monocytes is unclear. A limitation of our COC subgroup analyses is the small sample size; therefore, further studies are needed to evaluate the impact of COC use on diet-mediated changes in immune and inflammatory parameters.

Interestingly, we additionally observed correlations between changes in clinical immune cell subset counts and metabolic parameters, with the majority of associations observed with HDL parameters. We have previously demonstrated that HDL-C predicts clinical immune cell counts, where increases in HDL-C were associated with lower lymphocyte and monocyte counts in men and women, as well as lower neutrophil, eosinophil, and basophil counts in men [100]. These findings are consistent with our data, where changes in HDL-C, total HDL particles, and large and small HDL particle subsets negatively correlated with changes in clinical immune counts. HDL is an important modulator of immune cell activity, through modulation of lipid rafts and subsequent inflammation and cellular expansion in response to stimuli, carrying proteins with immunomodulatory properties, and by influencing cellular energy metabolism [53,108,109,110,111]. Accordingly, HDL profiles and its proteomic composition are modified and show therapeutic potential in acute and chronic inflammatory conditions such as CVD, autoimmune disorders, COVID-19, and sepsis [109,112]. Thus, further characterization of the immunomodulatory properties of HDL in response to egg intake is warranted. Conversely, we observed positive correlations between changes in clinical immune cell counts and metabolic parameters associated with chronic inflammatory disease risk, including serum triglycerides, triglyceride-rich lipoproteins, glucose, and LP–IR, suggesting that individuals who experienced increases in these metabolic parameters following whole egg intake were more likely to reflect immune profiles indicative of greater immune inflammation.

In addition to clinical leukocyte profiles, we examined the effects of egg intake on (1) erythrocyte markers, which serve as important indicators of anemia, inflammation, and nutritional status, and (2) platelets, which are essential for blood clotting and can serve as a marker of inflammation, as well as predictors of CVD and cancer risk [113,114,115]. Eggs contain various nutrients that support hematopoiesis and red blood cell profiles, including protein; vitamins A, D, and E; B vitamins; iron; calcium; manganese; magnesium; sodium and potassium; iodine; phosphorus; selenium; copper; zinc; and choline—many of which are contained in the egg yolk [4,5,116,117], which could explain the increase in hematocrit levels observed following the whole egg diet period. However, the effects of egg consumption on erythrocyte profiles and iron status—the most common nutritional cause of anemia and routine clinical indicators of red blood cell health [118]—remains somewhat controversial. Negative effects of egg intake on erythrocyte and iron status markers have been in part attributed to decreased iron bioavailability and limited effects on ferritin, which is responsible for iron-storage [118,119,120]. The effects of egg intake on iron status may be dependent on the fraction of eggs consumed, as yolk iron is predominantly contained within phosvitin, which is relatively resistant to digestion, thereby decreasing iron bioavailability [121,122], whereas iron contained within egg white-derived ovalbumin may promote non-heme iron absorption [122]. Egg consumption (whole or powdered) has been associated with reduced iron absorption—estimated by Hallberg and Hulthen to be ~27% for 3 eggs consumed [119], using an algorithm developed from observations from early feeding studies. While Leonard et al. [123] found that egg intake was weakly associated with lower serum ferritin (Spearman’s r = −0.28) in a cross-sectional study of 107 Australian women, additional recent studies have found no detrimental relationship between egg intake and iron status [124,125]. Six months of daily egg intake similarly did not alter iron or anemia status in young Malawian children [126]. In contrast, Little et al. [16] found that egg consumption was significantly correlated with reduced possibilities of mild anemia compared to subjects with no anemia, but no correlations were found in moderate and severe anemia subjects. For studies reporting improvements in erythrocyte profiles and iron status from whole egg intake such as ours, it is important to consider the role of choline given that serum choline levels were increased following the whole egg diet and choline deficiency in animals is associated with anemia [127]. Choline is also found to induce changes on red blood cell membrane properties, such as increasing membrane lipid fluidity [128], which might indirectly influence erythrocyte profiles. Further research is warranted to determine whether individual variability, overall nutritional quality of the diet, or nutrient composition/size of egg yolk and egg white fractions underlies the variable effects of egg intake on erythrocyte profiles.

We additionally observed a reduction in platelet counts following the egg white and whole egg period compared to the egg-free period. Various nutrients and dietary patterns additionally influence platelet parameters, where Mediterranean-style, plant-based, and omega-3- and antioxidant polyphenol-rich diet patterns and foods are associated with platelet marker benefits [129]. Interestingly, in a cohort study of 21,252 adults (≥20 years) from the Danish General Suburban Population Study (GESUS), Vinholt et al. [115] reported a U-shaped curve between mortality and platelet counts, with higher platelet counts within the normal range (100–450 × 10^9^/L) being associated with an increased risk of cardiovascular disease and increased risk of mortality with platelet counts over >300 × 10^9^/L. While average and individual platelet counts were within the normal range following each diet period, the reduction of platelet counts following the egg white and whole egg period relative to the egg white period—including participants with platelet counts >300 × 10^9^/L—is perhaps indicative of improved chronic disease risk [115].

## 5. Conclusions

In summary, we evaluated the effects of consuming different egg-based diets on comprehensive clinical parameters. Overall, the intake of whole eggs improved the nutrient density of the diet in various aspects while additionally improving choline status, HDL profiles, blood amino acid profiles indicative of T2DM risk (relative to egg white intake), hematocrit, and platelet counts. Our study additionally highlights unique lipid and immune responses to egg diets in females based on use of COC—a factor that is important to consider in diet intervention trials. Importantly, we observed strong associations between egg diet-induced changes in immune and HDL profiles, which warrant further investigation. Together, these findings suggest that, in a young healthy population, whole egg intake confers mostly beneficial changes in global clinical profiles.

## Figures and Tables

**Table 1:** Nutrient composition of daily egg treatment.

Nutrient	Egg White Diet	Whole Egg Diet
Total energy, kcal	75 ^1^	215.7
Total carbohydrates, g	3 ^1^	1.4
Total protein, g	15 ^1^	18.7
Total fat, g	0 ^1^	15
Cholesterol, mg	0 ^1^	621
Vitamin A, μg	0 ^1^	271.5
Vitamin C, mg	0 ^1^	0
Vitamin D, μg	0	3.7
Vitamin B2, mg	0.4	0.6
Choline, mg	0	507
Lutein + Zeaxanthin, μg	0	693
Selenium, μg	18.2	46.8
Sodium, mg	225 ^1^	194.7
Zinc, mg	0	1.9
Iron, mg	0 ^1^	2.5
Calcium, mg	0 ^1^	72.3

Nutrient composition of 3 large whole shelled eggs (whole egg diet) and the equivalent of 3 large egg whites (egg white diet), based on data retrieved from USDA FoodData Central database or ^1^ nutrient data from the nutrition facts label for the liquid egg white study food [38].

**Table 2:** Average daily nutrient intake of participants throughout egg diet periods.

Daily Intake Totals	Egg-Free Diet	Egg White Diet	Whole Egg Diet	*p*-Value
Total energy, kcal	1948.4 ± 817.7	1837.0 ± 633.2	2069.3 ± 586.7	0.240
Carbohydrates, % of kcal	49.5 ± 7.0 ^a^	45.6 ± 6.7 ^b^	41.8 ± 7.7 ^c^	<0.001
Protein, % of kcal	16.0 ± 4.4	18.3 ± 4.6	17.8 ± 3.9	0.062
Fat, % of kcal	32.2 ± 4.3 ^a^	34.8 ± 5.2 ^b^	39.4 ± 6.0 ^c^	<0.001
Total carbohydrates, g	244.1 ± 109.6	209.9 ± 75.9	221.6 ± 80.8	0.201
Total protein, g	77.4 ± 36.7	79.2 ± 25.1	88.0 ± 32.6	0.215
Animal protein, g	45.6 ± 26.4 ^a^	52.4 ± 19.2 ^ab^	58.4 ± 23.3 ^b^	0.023
Vegetable protein, g	31.8 ± 15.3	26.7 ± 10.2	29.5 ± 19.6	0.333
Alanine, g	3.45 ± 1.64	3.80 ± 1.26	4.12 ±1.54	0.080
Glycine, g	3.06 ± 1.38	3.21 ± 1.22	3.42 ± 1.36	0.390
Isoleucine, g	3.44 ± 1.71	3.71 ± 1.14	4.08 ± 1.50	0.101
Leucine, g	5.96 ± 2.88	6.23 ± 1.90	6.90 ± 2.50	0.145
Valine, g	3.88 ± 1.86	4.22 ± 1.21	4.56 ± 1.59	0.150
Total fat, g	71.6 ± 31.5 ^a^	75.8 ± 33.5 ^a^	92.1 ± 25.9 ^b^	0.003
Saturated fat, g	24.1 ± 11.3	25.1 ± 13.1	29.5 ± 8.3	0.058
Monounsaturated fat, g	24.7 ± 10.9 ^a^	25.9 ± 12.7 ^a^	32.6 ± 9.8 ^b^	0.001
Polyunsaturated fat, g	16.7 ± 9.2 ^a^	18.8 ± 9.5 ^ab^	21.4 ± 9.6 ^b^	0.035
Arachidonic acid, g	0.12 ± 0.13 ^a^	0.11 ± 0.06 ^a^	0.29 ± 0.08 ^b^	<0.001
EPA, g	0.03 ± 0.06	0.03 ± 0.05	0.04 ± 0.07	0.598
DHA, g	0.07 ± 0.13	0.06 ± 0.11	0.12 ± 0.21	0.245
Alcohol, g	6.1 ± 8.6	3.7 ± 6.1	4.0 ± 6.8	0.084
Cholesterol, mg	224.0 ± 286.6 ^a^	170.1 ± 89.4 ^a^	657.8 ± 125.1 ^b^	<0.001
Vitamin A, μg	1001.1 ± 615.1	1036.5 ± 605.8	1156.1 ± 569.4	0.501
Pantothenic acid, mg	5.0 ± 2.5 ^ab^	4.2 ± 1.4 ^a^	5.9 ± 1.5 ^b^	0.001
Vitamin B6, mg	1.9 ± 0.8	1.7 ± 0.8	2.0 ± 1.3	0.377
Folic acid, μg	475.9 ± 270.7	385.4 ± 144.6	551.5 ± 667.2	0.298
Vitamin B12, μg	3.72 ± 2.7 ^ab^	3.0 ± 1.8 ^a^	4.8 ± 2.7 ^b^	0.016
Vitamin C, mg	88.2 ± 63.1	75.8 ± 42.0	90.0 ± 62.5	0.440
Vitamin D, μg	4.2 ± 4.4 ^ab^	3.1 ± 2.6 ^a^	6.1 ± 2.7 ^b^	0.006
Vitamin E, mg α-tocopherol	11.9 ± 8.7	10.5 ± 6.5	13.5 ± 7.0	0.239
Vitamin K, μg	107.8 ± 74.4	142.7 ± 81.2	141.7 ± 106.9	0.113
Calcium, mg	919.5 ± 471.8	795.9 ± 314.1	910.7 ± 355.4	0.293
Copper, mg	1.26 ± 0.65	1.12 ± 0.49	1.14 ± 0.59	0.216
Iron, mg	15.8 ± 8.0	12.0 ± 5.0	16.1 ± 14.1	0.204
Magnesium, mg	300.4 ± 140.2	260.0 ± 106.1	264.0 ± 125.0	0.075
Phosphorus, mg	1197.8 ± 510.9 ^ab^	1042.8 ± 375.1 ^a^	1304.5 ± 454.0 ^b^	0.020
Selenium, μg	111.4 ± 61.3 ^a^	117.4 ± 36.0 ^a^	143.1 ± 43.8 ^b^	0.017
Sodium, mg	3170.7 ± 1146.4 ^a^	3387.0 ± 1238.6 ^ab^	3862.5 ± 1328.8 ^b^	0.032
Zinc, mg	10.4 ± 4.4	8.8 ± 3.8	11.1 ± 6.5	0.127
Choline, mg	289.5 ± 253.1 ^a^	221.2 ± 78.4 ^a^	614.1 ± 147.2 ^b^	<0.001
Betaine, mg	180.4 ± 150.5	126.9 ± 60.2	143.1 ± 80.3	0.155
Lutein + Zeaxanthin, μg	1425.1 ± 1007.2 ^a^	2063.0 ± 1901.5 ^ab^	2471.6 ± 2208.1 ^b^	0.039

Data are reported as mean ± standard deviation, n = 26. Values with different letters (a, b, c) are significantly different at *p* < 0.05.

**Table 3:** Anthropometric and serum metabolic profiles following egg diet periods.

	Egg-Free Diet	Egg White Diet	Whole Egg Diet	*p*-Value
Body weight, kg	66.7 ± 12.1 ^a^	67.3 ± 12.7 ^b^	67.2 ± 12.7 ^ab^	0.038
BMI, kg/m^2^	22.9 ± 2.8	23.0 ± 3.0	23.0 ± 3.0	0.054
Body fat, %	24.8 ± 7.1	25.5 ± 7.3	26.1 ± 7.7	0.189
Fat mass, g	36.9 ± 15.1 ^a^	37.7 ± 15.9 ^ab^	38.2 ± 16.2 ^b^	0.020
Fat free mass, g	109.8 ± 20.1	110.3 ± 21.0	109.6 ± 20.3	0.241
Muscle mass, g	104.0 ± 19.5	104.5 ± 20.3	102.8 ± 20.7	0.142
Sodium, mmol/L	138.8 ± 1.6	139.2 ± 1.5	139.0 ± 1.8	0.623
Potassium, mmol/L	4.13 ± 0.24	4.11 ± 0.23	4.11 ± 0.26	0.913
Chloride, mmol/L	104.0 ± 1.8	104.7 ± 2.0	104.1 ± 1.8	0.248
Carbon dioxide, mmol/L	23.9 ± 3.0	23.9 ± 2.4	23.8 ± 2.5	0.977
Calcium, mg/dL	9.35 ± 0.29	9.32 ± 0.30	9.35 ± 0.24	0.921
BUN, mg/dL	12.7 ± 3.2	13.0 ± 3.0	12.9 ± 3.0	0.907
Protein, g/dL	0.807 ± 0.09	0.784 ± 0.13	0.78 ± 0.11	0.160
Albumin, g/dL	4.42 ± 0.30	4.43 ± 0.26	4.40 ± 0.23	0.735
Globulin, g/dL	2.41 ± 0.32	2.42 ± 0.33	2.52 ± 0.34	0.170
Albumin:Globulin	1.88 ± 0.33	1.86 ± 0.30	1.78 ± 0.25	0.190
Bilirubin, mg/dL	0.53 ± 0.25	0.55 ± 0.33	0.50 ± 0.2	0.681
ALP, U/L	55.9 ± 14.1	55.9 ± 17.0	54.5 ± 11.9	0.983
ALT, U/L	14.4 ± 6.1	13.0 ± 6.8	13.2 ± 5.1	0.453
AST, U/L	18.5 ± 5.5	17.9 ± 5.2	18.0 ± 4.5	0.751
hsCRP, mg/L	1.5 ± 1.9	1.7 ± 3.1	2.2 ± 3.8	0.629
Total ketones, μmol/L	170.9 ± 105.3	167.2 ± 141.4	137.4 ± 54.6	0.468
BHB, μmol/L	173.7 ± 106.6	170.1 ± 143.7	138.6 ± 55.4	0.462
Acetoacetate, μmol/L	39.8 ± 21.7	40.4 ± 29.6	38.0 ± 16.0	0.934
Acetone, μmol/L	36.3 ± 16.3	34.3 ± 15.5	31.5 ± 11.8	0.482
Choline, μM	8.8 ± 2.8 ^a^	9.6 ± 2.3 ^ab^	10.6 ± 2.6 ^b^	0.025
Betaine, μM	32.4 ± 10.4 ^a^	31.6 ± 10.9 ^a^	36.6 ± 13.4 ^b^	<0.001
TMAO, μM	2.1 ± 1.5	4.0 ± 6.4	1.6 ± 0.9	0.092

Data are reported as mean ± standard deviation, n = 23–26. Values with different letters (a, b) are significantly different at *p* < 0.05. ALP: alkaline phosphatase; ALT: alanine aminotransferase; AST: aspartate aminotransferase; BHB: beta-hydroxybutyrate; BMI: body mass index; BUN: blood urea nitrogen; TMAO: trimethylamine *N*-oxide.

**Table 4:** Serum insulin sensitivity markers following egg diet periods.

	Egg-Free Diet	Egg White Diet	Whole Egg Diet	*p*-Value
Glucose, mg/dL	81.6 ± 9.6	82.5 ± 9.4	83.5 ± 9.4	0.423
GlycA, μmol/L	380.2 ± 75.1	367.1 ± 44.7	386.9 ± 79.7	0.333
LP–IR risk index, 0–100	34.1 ± 16.6	33.4 ± 15.9	31.4 ± 13.6	0.701
DRI, 0–100	32.7 ± 11.7	32.8 ± 10.3	33.4 ± 10.1	0.947
Total BCAA, μmol/L	361.9 ± 44.1	376.1 ± 50.8	384.1 ± 46.5	0.079
Leucine, μmol/L	123.4 ± 20.0	120.9 ± 24.8	126.4 ± 19.0	0.612
Isoleucine, μmol/L	50.4 ± 10.6 ^a^	56.9 ± 9.5 ^b^	58.4 ± 11.1 ^b^	0.001
Valine, μmol/L	188.0 ± 28.5	198.3 ± 31.5	199.2 ± 30.6	0.133
Alanine, μmol/L	363.4 ± 98.0	365.5 ± 83.0	378.7 ± 65.9	0.608
Glycine, μmol/L	202.9 ± 53.2 ^a^	196.0 ± 47.7 ^a^	219.2 ± 61.2 ^b^	0.004
Citrate, μmol/L	86.0 ± 24.4	87.6 ± 24.9	81.3 ± 19.2	0.408

Data are reported as mean ± standard deviation, n = 25–26. Values with different letters (a, b) are significantly different at *p* < 0.05. BCAA: branched-chain amino acids; DRI: diabetes risk index; LP–IR: lipoprotein insulin resistance risk index.

## Data Availability

Data may be made available upon reasonable request by contacting the corresponding author.

## Supplementary Materials

The following supporting information can be downloaded at: https://www.mdpi.com/article/10.3390/nu15173747/s1, Table S1: Baseline characteristics of study participants; Table S2: Dietary intake of food groups across diet periods; Table S3: Serum lipoprotein profiles following egg diet periods; Table S4: Clinical immune profiles following egg diet periods.
