# Supplementary material for: Consumption of Different Egg-Based Diets Alters Clinical Metabolic and Hematological Parameters in Young, Healthy Men and Women
Source: Nutrients. 2023 Aug 27;15(17):3747. doi: 10.3390/nu15173747 (PMC10490185; doi:10.3390/nu15173747)
Supplement: Supplementary file 1 [file nutrients-15-03747-s001.zip › nutrients-2514188-supplementary.pdf]

Table S1. Baseline characteristics of study participants

Baseline Characteristics	
Age (years)	21.7 ± 4.7
Sex	
Male (n)	5
Female (n)	21
BMI (kg/m^2)	22.5 ± 2.8
Body fat percentage (%)	24.8 ± 7.1
Physical activity (min/day)	78.7 ± 59.8
Smoking (n)	
Yes	2
No	24
Medications (n)	
Combined oral contraceptives (birth control)	13
Mood stabilizers	5
Asthma	3
Allergy	2
Beta blockers	2
Acne treatment	1
Laxative	1
Dietary Supplements (n)	
Multivitamin	7
Iron	1
Vitamin C	1
Probiotic	1

Data are presented as mean ± standard deviation or total number of participants (n). n = 26.

Table S2. Average dietary intake of food groups across diet periods.

Food Group (servings per day)	Egg-free diet	Egg white diet	Whole egg diet
Vegetables	2.91 ± 1.75	3.32 ± 1.76	3.21 ± 2.51
Fruits	1.32 ± 1.23	1.00 ± 0.89	1.18 ± 1.03
Grains	8.34 ± 7.39	5.75 ± 2.30	6.52 ± 3.48
Dairy	2.03 ± 1.34	1.97 ± 1.41	2.08 ± 1.26
Protein foods	4.54 ± 2.72 [a]	6.51 ± 3.59 [b]	7.16 ± 3.56 [b]
Oils	2.62 ± 1.46 [a]	3.72 ± 2.32 [b]	4.48 ± 2.22 [b]

Data are presented as mean ± standard deviation, n = 26. Values with different letters (a, b) are significantly different at $p < 0.05$.

Table S3. Serum lipoprotein profiles following egg diet periods.

	**Egg-free diet**	**Egg white diet**	**Whole egg diet**	***p*-value**
Total TRLP particles, nmol/L	83.8 ± 33.0	91.2 ± 57.0	85.5 ± 55.5	0.824
Average TRLP particle size, nm	48.1 ± 8.6	48.8 ± 7.9	48.1 ± 6.6	0.896
Very large TRLP, nmol/L	0.14 ± 0.18	0.20 ± 0.20	0.17 ± 0.11	0.463
Large TRLP, nmol/L	1.7 ± 1.9	1.1 ± 1.1	1.5 ± 1.9	0.494
Medium TRLP, nmol/L	16.9 ± 10.4	16.5 ± 12.4	18.9 ± 13.3	0.556
Small TRLP, nmol/L	20.1 ± 23.7	10.8 ± 10.6	14.8 ± 18.1	0.116
Very Small TRLP, nmol/L	45.0 ± 31.9	62.6 ± 54.3	50.1 ± 48.5	0.379
Total LDL particles, nmol/L	1,144.7 ± 275.1	1,176.0 ± 272.7	1,213.8 ± 230.7	0.190
Average LDL particle size, nm	21.5 ± 0.3	21.4 ± 0.5	21.4 ± 0.4	0.326
Large LDL, nmol/L	478.6 ± 126.8	461.4 ± 181.2	492.1 ± 165.2	0.737
Medium LDL, nmol/L	422.9 ± 203.9	381.4 ± 204.9	362.6 ± 244.0	0.388
Small LDL, nmol/L	243.2 ± 148.3	333.2 ± 280.8	359.1 ± 231.9	0.189
Total HDL particle number, µmol/L	20.2 ± 2.4	20.6 ± 2.7	20.8 ± 3.3	0.482
Average HDL particle size, nm	9.3 ± 0.4	9.3 ± 0.4	9.3 ± 0.3	0.629
Large HDL, µmol/L	2.2 ± 1.4	2.5 ± 1.4	2.6 ± 1.6	0.244
Medium HDL, µmol/L	6.5 ± 2.8	6.0 ± 2.8	6.1 ± 2.6	0.435
Small HDL, µmol/L	11.5 ± 2.6	12.2 ± 2.9	12.1 ± 3.0	0.475
Serum apoB, mg/dL	67.4 ± 15.5	68.1 ± 15.2	69.9 ± 12.3	0.483
Serum apoA1, mg/dL	137.3 ± 20.8	140.4 ± 21.9	143.9 ± 25.7	0.189

Data are reported as mean ± standard deviation, n = 25. ApoA-1: apolipoprotein A-1; apoB: apolipoprotein B; TRLP: triglyceride-rich lipoprotein.

Table S4. Clinical immune profiles following egg diet periods.

	**Egg-free diet**	**Egg white diet**	**Whole egg diet**	***p*-value**
Total WBC,1000 cells/μl	5.81 ± 1.15	5.53 ± 1.24	5.68 ± 1.33	0.521
Neutrophils, 1000 cells/μl	3.17 ± 1.06	3.01 ± 0.97	2.97 ± 1.04	0.591
Lymphocytes, 1000 cells/μl	2.01 ± 0.47	1.88 ± 0.40	2.04 ± 0.44	0.255
Monocytes, 1000 cells/μl	0.48 ± 0.10	0.45 ± 0.13	0.49 ± 0.11	0.234
Eosinophils, 1000 cells/μl	0.15 ± 0.10	0.20 ± 0.29	0.14 ± 0.07	0.340
Basophils, 1000 cells/μl	0.04 ± 0.02	0.04 ± 0.02	0.05 ± 0.02	0.743
% Neutrophils, % of total WBC	53.4 ± 8.8	53.1 ± 8.9	50.9 ± 7.6	0.415
% Lymphocytes, % of total WBC	34.4 ± 7.2	34.6 ± 6.5	36.5 ± 6.8	0.391
% Monocytes, % of total WBC	8.4 ± 2.0	8.3 ± 2.0	8.8 ± 1.5	0.365
% Eosinophils, % of total WBC	2.7 ± 2.3	3.7 ± 4.9	2.6 ± 1.4	0.259
% Basophils, % of total WBC	0.7 ± 0.3	0.8 ± 0.3	0.8 ± 0.3	0.600
Neutrophil:lymphocyte ratio	1.70 ± 0.82	1.61 ± 0.52	1.49 ± 0.51	0.485
Lymphocyte:monocyte ratio	4.30 ± 1.29	4.41 ± 1.10	4.28 ± 1.05	0.866

Data are reported as mean ± standard deviation. WBC: white blood cells.
